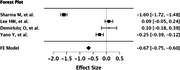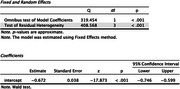# Pentraxin 3 and Cognitive Impairment: An Updated Systematic Review and Meta‐Analysis

**DOI:** 10.1002/alz70856_098329

**Published:** 2025-12-24

**Authors:** Elizabeth Divina, Rani Permata, Kaven Tannardi, Pukovisa Prawiroharjo

**Affiliations:** ^1^ Faculty of Medicine, Universitas Indonesia, Central Jakarta, Jakarta, Indonesia; ^2^ Department of Neurology, Faculty of Medicine, Universitas Indonesia, Jakarta, Indonesia, Central Jakarta, Jakarta, Indonesia; ^3^ Dr. Cipto Mangunkusumo Public Hospital, Central Jakarta, DKI Jakarta, Indonesia

## Abstract

**Background:**

Pentraxin‐3 (PTX3) emerges as a promising biomarker for cognitive impairment due to its association wit**h inflammatory processes implicated in vascular pathologies. Given that cognitive impairment often manifests as a consequence of vascular events, PTX3's ability to reflect vascular inflammation makes it a relevant candidate for assessing cognitive decline in such contexts. This is an updated systematic review and the first meta‐analysis between PTX3 and cognitive function**.

**Method:**

A systematic review was conducted using five databases (Pubmed and MEDLINE, Proquest, Scopus, Science Direct, Cochrane) and individual searches on 02 January 2025. This review included those that studies adult patients undergoing cognitive assessment.

**Result:**

After removing duplicates, we obtained 1512 articles. After undergoing abstract and full‐text screening, this study discussed 8 articles, out of which, 1 article was a systematic review. From the selected studies, the combined sample population was 6,004 people and the average combined age was 69.08 ± 11.65 years old. Three studies used Modified Mini Mental Examination (3MSE) to measure cognitive function and the average combined score was 91.425 ± 4.90 while four studies using Mini Mental State Examination (MMSE) had the average combined score 25.2975 ± 3.56, both which are considered normal. The average combined value of PTX3 was 1.070364 ± 0.9657 ng/mL. From 4 studies, a meta analysis of the correlation between PTX3 and 3MSE or MMSE scores found a significant negative correlation (r=‐0.67; *p* <0.001). One study found that PTX3 increases cognitive decline by 20% (*p* <0.0025), another found that PTX3 had significant negative correlation with executive function domain (r=‐0.057; *p* = 0.05). Last but not least, one study found that while PTX3 had significant effect on cognitive decline in women, PTX3 was found to be a protective factor against cognitive decline in men.

**Conclusion:**

Our study suggests that PTX3 had a significant negative correlation towards cognitive impairment measured by 3MSE or MMSE, therefore it has a potential to be a novel less‐invasive biomarker to predict cognitive function. Studies correlating PTX3 and cognitive function are still limited and warrants further research.